# Safety and efficacy of ixoberogene soroparvovec in neovascular age-related macular degeneration in the United States (OPTIC): a prospective, two-year, multicentre phase 1 study

**DOI:** 10.1016/j.eclinm.2023.102394

**Published:** 2023-12-22

**Authors:** Arshad M. Khanani, David S. Boyer, Charles C. Wykoff, Carl D. Regillo, Brandon G. Busbee, Dante Pieramici, Carl J. Danzig, Brian C. Joondeph, James C. Major, Adam Turpcu, Szilárd Kiss

**Affiliations:** a Sierra Eye Associates, Reno, NV, USA; b The University of Nevada, Reno School of Medicine, Reno, NV, USA; c Retina Vitreous Associates Medical Group, Beverly Hills, CA, USA; d Retina Consultants of Texas, Retina Consultants of America, Blanton Eye Institute, Houston Methodist Hospital, Houston, TX, USA; e Mid Atlantic Retina, Wills Eye Hospital, Thomas Jefferson University, Philadelphia, PA, USA; f Tennessee Retina, Nashville, TN, USA; g California Retina Consultants, Bakersfield, CA, USA; h Rand Eye Institute, Deerfield Beach, FL, USA; i Florida Atlantic University, Charles E. Schmidt School of Medicine, Boca Raton, FL, USA; j Colorado Retina Associates, Denver, CO, USA; k Adverum Biotechnologies, Redwood City, CA, USA; l Department of Ophthalmology, Weill Cornell Medical College, New York-Presbyterian Hospital, New York, NY, USA

**Keywords:** Neovascular age-related macular degeneration, Gene therapy, Anti-VEGF, Ixoberogene soroparvovec

## Abstract

**Background:**

Gene therapy, successfully used in rare, monogenetic disorders, may prove to be a durable management approach for common, polygenetic conditions, including neovascular age-related macular degeneration (nAMD). Repeated injections, oftentimes monthly, and possibly for decades, of vascular endothelial growth factor antagonists (anti-VEGF), is the standard for nAMD. We hypothesised that an in-office, intravitreal administration of ixoberogene soroparvovec (ixo-vec, formerly ADVM-022), a single-dose gene therapy encoding for the proven anti-VEGF protein, aflibercept, would transform retinal cells to continually produce aflibercept to minimise treatment burden in nAMD.

**Methods:**

In this two-year, open-label, prospective, multicentre phase 1 study, patients with nAMD responding to anti-VEGF were assigned to four cohorts differing by ixo-vec dose (2 × 10^11^ vs 6 × 10^11^ vector genomes (vg/eye)) and prophylactic steroids (oral prednisone vs topical difluprednate). The primary outcome was the type, severity, and incidence of ocular and systemic adverse events (AEs); secondary endpoints included vision, central subfield thickness (CST), and the number of supplemental injections. This study was registered with ClinicalTrials.gov, NCT03748784.

**Findings:**

Thirty patients with nAMD were enrolled between November 14, 2018 and June 30, 2020 at nine study sites in the United States. No systemic ixo-vec related AEs were noted. Across both dose groups the most common adverse event was anterior chamber cell, which was reported in 11 participants in the 6 × 10^11^ dose group and in 7 participants in the 2 × 10^11^ dose group; intraocular inflammation was responsive to topical corticosteroids, with no anterior chamber cells or vitreous cells observed in 2 × 10^11^ vg/eye patients at the end of the study. Vision and CST remained stable throughout two years with annualised anti-VEGF injections reduced by 80% (10.0 mean annualised anti-VEGF injections to 1.9) in 2 × 10^11^ vg/eye and 98% (9.8 mean annualised anti-VEGF injections to 0.2) in 6 × 10^11^ vg/eye cohorts.

**Interpretation:**

Ixo-vec was generally well-tolerated, maintained vision, and improved anatomical outcomes in nAMD, with a substantial reduction in anti-VEGF injections. A single administration of an in-office gene therapy, with vectorised protein with an already established clinical benefit, has the potential to revolutionise the management of common ocular disorders requiring ongoing, frequent therapeutic interventions.

**Funding:**

Adverum Biotechnologies.

**Research in context**

**Evidence before this study**

On October 27, 2023, we conducted a search of the PubMed database for articles with the terms “gene therapy” (all fields) and “age related macular degeneration” (title/abstract fields) with reviews excluded. We identified 60 articles, of which seven reported results of clinical trials evaluating gene therapies for nAMD. Of these seven, five were studies of rAAV.sFLT-1, a recombinant adeno-associated vector encoding sFLT-1, which is delivered via subretinal injection with pars plana vitrectomy. One study evaluated intravitreous injection of AAV2-sFLT01, an AAV2 vector that expresses VEGF-neutralising protein sFLT01. One study evaluated intravitreous delivery of an E1-, partial E3-, E4-deleted adenoviral vector expressing human pigment epithelium-derived factor (AdPEDF.11).

**Added value of this study**

To the best of our knowledge, ixo-vec is the first ocular gene therapy for nAMD to be successfully administered intravitreally that results in stable and durable expression of aflibercept, a proven therapeutic anti-VEGF. The results of the OPTIC study provide further support for intravitreal gene therapy as a potential treatment option for nAMD.

**Implications of all the available evidence**

In this 2-year, prospective, open-label study, Ixo-vec was generally well tolerated and substantially reduced the need for further anti-VEGF injections in treatment-experienced patients with nAMD, with over half of the patients requiring no additional anti-VEGF treatment. Further investigation of ixo-vec in patients with nAMD is warranted in larger, randomised controlled studies.

## Introduction

After nearly fifty years of advances, *ex-vivo* and *in-vivo* gene therapies have brought viable treatment options to patients with an assortment of cancers and inherited genetic diseases.^1^ In the United States, the era of gene therapy began in 2017 following Food and Drug Administration (FDA) approvals of *ex-vivo* gene therapy with chimeric antigen receptor (CAR)-T cells to treat B cell malignancies and adeno-associated virus (AAV) based *in-vivo* gene therapy to treat congenital blindness, specifically RPE65-associated Leber congenital amaurosis.^1^^,^^2^ Due to its relative immune privilege, the eye represents an ideal environment to deliver viral vector-based *in vivo* gene therapy via intravitreal, subretinal, or suprachoroidal delivery.^2^^,^^3^ Gene therapy for retinal disease is especially promising since most inherited retinal disorders (IRDs) are monogenetic, cells are post-mitotic, and the imaging modalities used for diagnosis and monitoring are well-described and non-invasive.^2^^,^^3^

The durable, possibly curative, benefit of a single administration of gene therapy that is employed to target rare IRDs, may also be used to treat common, non-inherited retinal diseases affecting millions of people, such as age-related macular degeneration (AMD).^2^^,^^3^ Rather than correcting for a specific genetic alteration as is done for IRDs, a novel approach for AMD would utilise vector-mediated introduction of a genetic sequence (i.e., biofactory approach) to generate endogenous production of therapeutic proteins targeting well-established pathways, such as vascular endothelial growth factor (VEGF) antagonists.^2^

AMD is one of the leading causes of irreversible visual impairment in individuals 50 years or older.^4^^,^^5^ Neovascular AMD (nAMD) is an advanced form of AMD, accounting for 10–20% of AMD cases and is responsible for 80–90% of severe visual loss in this disorder.^5^^,^^6^ VEGF is a key mediator of the pathological angiogenesis and the formation of macular choroidal neovascularisation (CNV) that, without intervention, is directly responsible for vision loss in nAMD.^2^^,^^4^^,^^5^^,^^7^ Repeated intravitreal bolus injections of VEGF inhibitors (anti-VEGF) can inhibit CNV growth and leakage and stabilise or improve vision outcomes in patients with nAMD.^8^^,^^9^ Aflibercept is an anti-VEGF recombinant fusion protein, which was FDA approved for nAMD in 2011 and has been administered intravitreally over 20 million times worldwide to treat VEGF-mediated retinal disorders.^3^^,^^7^

While anti-VEGF intravitreal injections, such as aflibercept, revolutionised nAMD treatment, current therapies necessitate frequent injections and monitoring as often as every 4 weeks for the lifetime of the patient, although treat-and-extend regimens have enabled longer treatment duration for many patients.^4^^,^^8^ Further, real world visual outcomes do not match those of clinical trials.^8^^,^^10^^,^^11^ This is due in part to the considerable treatment burden of frequent injections and significant undertreatment in many real-world settings, with patients in some studies receiving fewer than half the annual recommended number of intravitreal injections.^4^^,^^12^^,^^13^ Furthermore, recent research suggests that intermittent bolus therapy and associated retinal fluid volatility may lead to long term visual loss.^14^^,^^15^

To improve clinical outcomes and decrease treatment burden in nAMD, we pursued an *in-vivo* genotype-independent biofactory approach in which a gene therapy, ixoberogene soroparvovec (ixo-vec, formerly ADVM-022), is delivered via a single, in-office, intravitreal injection, resulting in the long term, stable expression of a proven therapeutic anti-VEGF, aflibercept.^2^^,^^3^

Ixo-vec utilises a novel vector capsid, AAV2.7m8, carrying an aflibercept coding sequence under the control of a ubiquitous expression cassette (Figure S1).^4^ AAV2.7m8 was engineered from AAV2 via directed evolution in rodents, canines and nonhuman primates and includes a 10-amino acid insertion in loop IV of the AAV2 viral structural spike proteins (VP1-3), which has been shown to facilitate transit across the inner limiting membrane (ILM).^4^^,^^16^^,^^17^ In preclinical studies, ixo-vec administration resulted in long-term, stable expression of aflibercept at levels expected to be adequate to treat nAMD with no measurable effect on normal retinal structure or function observed following long-term VEGF suppression.^4^^,^^8^^,^^12^

This prospective, two-year, multicentre phase 1 study (OPTIC) assessed the safety and efficacy of a single intravitreal injection of ixo-vec in patients with nAMD previously controlled with frequent anti-VEGF injections.

## Methods

### Study design

**Fig. 1:**
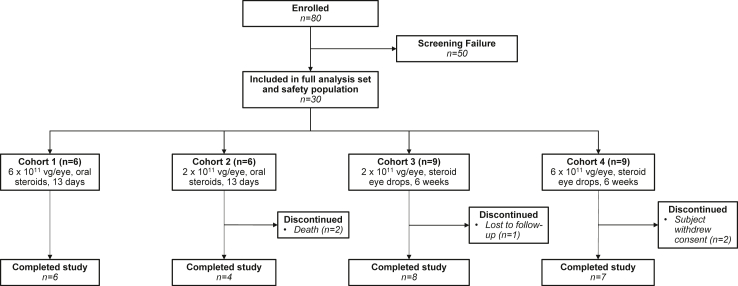
Patient disposition.

This prospective, open-label, dose-ranging, phase 1 study (NCT03748784) enrolled 30 patients with nAMD who required regular intravitreal anti-VEGF injections between November 14, 2018 and June 30, 2020 at nine study sites in the United States (Fig. 1). Eligible participants were sequentially enrolled to one of four cohorts: six participants each to cohorts 1 and 2 and nine participants each to cohorts 3 and 4. Cohorts 1 and 4 received the high dose of ixo-vec, 6 × 10^11^ vg/eye, and cohorts 2 and 3 received the low dose of ixo-vec, 2 × 10^11^ vg/eye (Figure S2). Cohorts 1 and 2 followed a 13-day prophylactic oral prednisone regimen. Cohorts 3 and 4 followed a 6-week prophylactic topical difluprednate regimen. Participants were followed for two years with an optional three-year extension study (NCT04645212).

Eligible participants were males or females ≥50 years of age with current or prior evidence of active subfoveal CNV secondary to AMD occupying ≥50% of total lesion size with leakage on fluorescein angiography (FA), fluid on SD-OCT, or subretinal hemorrhage on color fundus photo. Participants must have had active anti-VEGF treatment for nAMD with a minimum of 2 injections within 4 months prior to study screening. Best corrected visual acuity (BCVA) in the study eye at screening had to be between 78 and 25 Early Treatment Diabetic Retinopathy Study (ETDRS) letters, inclusive (approximate Snellen equivalent 20/32 to 20/320). Finally, participants had to have demonstrated a meaningful anti-VEGF response during screening, defined as a reduction in central subfield thickness (CST) on SD-OCT of ≥30% from initial diagnosis or ≥ 20% from screening, or the normalisation of CST with no nAMD activity. Exclusion criteria were: 1) documented neutralising anti-AAV.7m8 antibody titer levels >1:125 within 6 months prior to dosing; 2) evidence of CNV lesion characteristics preventing improvements in visual acuity: scarring, atrophy, fibrosis, or subretinal hemorrhage comprising ≥50% of total lesion area; or blood under the fovea ≥1 disc area in size; and 3) various medical history criteria, including ocular and non-ocular conditions. Only one eye per participant was selected as the study eye; if both eyes meet all inclusion/exclusion criteria, the eye with the worst BCVA assessed at Screening was selected as the study eye.

At screening, prospective participants received a single dose of aflibercept 2 mg intravitreally consistent with the standard-of-care and underwent clinical evaluation 7–15 days later (i.e., study Day 1) to determine study eligibility including meaningful anti-VEGF responses which were confirmed by the Investigator and the Sponsor. Eligible participants then received ixo-vec intravitreal administration on Day 1 and returned for clinical evaluation on Days 3 and 8, then at Weeks 2, 4, 6, 8, and every 4 weeks thereafter through Week 104. Supplemental aflibercept was initiated as early as Week 4 if any of the following criteria were met: loss of ≥10 letters in BCVA from baseline attributed to intraretinal or subretinal fluid, increase in CST >75 μm from baseline, or presence of vision-threatening hemorrhage due to AMD; subsequent rescues were administered according to standard-of-care. Record review and documentation of all prior anti-VEGF use during the year prior for enrolled participants was performed under a separate protocol.

### Endpoints

Primary endpoints were the type, severity, and incidence of ocular and systemic adverse events (AEs). Secondary endpoints included the mean change in BCVA from baseline (baseline measurements were the values at screening), mean change from baseline in CST and macular volume over time, mean number of supplemental aflibercept injections over time as compared with the mean annualised anti-VEGF use in the year prior, and percentage of participants requiring supplemental aflibercept injections over time.

### Ethics

The study was performed in accordance with the ethical principles of the Declaration of Helsinki and Council of International Organisations of Medical Sciences and the International Council for Harmonisation of Technical Requirements for Pharmaceuticals for Human Use guidelines. Study protocols were approved by the Institutional Review Board/Institutional Biosafety Committee, and written informed consent was obtained from all participants. Participant safety was overseen by an independent Data Monitoring Committee.

### Statistical analysis

The safety population included all participants who received ixo-vec and was analysed according to cohort, dose received, and in aggregate. No formal calculations were performed to determine the sample size. AEs were coded using MedDRA classification.

Descriptive statistics were used to assess efficacy by cohort, dose received, and in aggregate. Vision was assessed through BCVA expressed as an ETDRS score. Spectral Domain Ocular Coherence Tomography (SD-OCT) was conducted on the study eye at all visits to assess CST. Imaging was performed prior to administering aflibercept at any scheduled visits. The observed value and change from Baseline for both BCVA and CST endpoints were summarised using continuous descriptive statistics and 90% confidence intervals by visit (including the derived ‘last visit’ time point). Confidence intervals were calculated using the t-distribution. Statistical programming and analyses were performed using SAS® Version 9.4 or higher.

### Role of the funding source

Adverum Biotechnologies participated in study design; collection, analysis, and interpretation of data; in the writing of the report; and in the decision to submit for publication. All authors had full access to all the data in the study and accept responsibility for the decision to submit for publication.

## Results

### Participant characteristics

Thirty participants aged 62–90 years were enrolled in the study. Baseline characteristics were generally comparable across the 4 cohorts although higher baseline CST values were reported in cohort 3 compared with the other cohorts (Table 1). The mean annualised number of anti-VEGF injections in the year prior to the study ranged from 9.6 to 10.5, indicating a substantial treatment burden for participants with active nAMD.

**Table 1:** Baseline characteristics of the participants.

Baseline characteristics^a^	Cohort 1 6 × 10^11^ (N = 6)	Cohort 2 2 × 10^11^ (N = 6)	Cohort 3 2 × 10^11^ (N = 9)	Cohort 4 6 × 10^11^ (N = 9)
Age, years				
Mean (SD)	79.0 (9.57)	79.8 (6.21)	77.4 (8.35)	79.9 (5.99)
Sex: n (%)				
Male	5 (83%)	3 (50%)	3 (33%)	4 (44%)
Female	1 (17%)	3 (50%)	6 (67%)	5 (56%)
Race: n (%)				
American Indian or Alaska Native	0 (0%)	0 (0%)	0 (0%)	0 (0%)
Asian	0 (0%)	0 (0%)	0 (0%)	0 (0%)
Black or African American	0 (0%)	0 (0%)	0 (0%)	0 (0%)
Native Hawaiian or Other Pacific Islander	0 (0%)	0 (0%)	0 (0%)	0 (0%)
White	6 (100%)	6 (100%)	9 (100%)	9 (100%)
Multi-Racial	0 (0%)	0 (0%)	0 (0%)	0 (0%)
Other	0 (0%)	0 (0%)	0 (0%)	0 (0%)
Years Since nAMD Diagnosis				
Median (IQR)	3.6 (1.1, 7.1)	4.4 (2.0, 6.3)	2.2 (1.3, 4.6)	3.5 (0.6, 5.2)
Anti-VEGF Injections Since Initial Diagnosis^b^				
Median (IQR)	29.0 (9.0, 46.0)	34.5 (17.0, 45.0)	21.0 (11.0, 25.0)	14.0 (4.0, 48.0)
Anti-VEGF Injections in 12 Months Prior to Screening				
Mean (SD)	9.2 (0.98)	9.2 (1.83)	8.9 (0.93)	6.6 (2.60)
Annualised anti-VEGF Injections in 12 months Prior to Ixo-vec				
Mean (SD)	9.7 (1.03)	10.5 (1.11)	9.6 (1.43)	9.9 (2.97)
BCVA, ETDRS Letters				
Mean (SD)	65.8 (6.91)	64.7 (7.55)	65.9 (7.64)	65.0 (7.78)
Approximate Snellen Equivalent	20/50	20/50	20/50	20/50
CST, μm				
Mean (SD)	369.2 (98.72)	307.7 (38.21)	473.4 (196.93)	398.6 (96.71)

a Median and IQR defined as (Q1, Q3) are provided for description of variables with a skewed distribution.

b Not including the mandated aflibercept at Screening.

### Safety outcomes and primary endpoint

The majority of ixo-vec related ocular treatment-emergent adverse events (TEAEs) were dose-dependent and mild (118/141, 83.7%) to moderate (22/141, 15.6%) in severity. The most reported ixo-vec related ocular TEAEs were anterior chamber cell in 16 participants (10 in the 6 × 10^11^ dose group and 6 in the 2 × 10^11^ dose group) and vitreal cells in 11 participants (8 in the 6 × 10^11^ dose group and 3 in the 2 × 10^11^ dose group) (Table S1). Five serious TEAEs (SAEs) were reported: two cases of cataract, dry AMD, retinal detachment, and a case of recurrent uveitis (Table 2). Two of the five reported SAEs were deemed to be probably related to ixo-vec: dry AMD, where there was asymmetric progression of pre-existing dry AMD, and recurrent uveitis, where uveitis recurred following the discontinuation of corticosteroid therapy for inflammation. The unrelated ocular SAE of retinal detachment occurred 301 days after ixo-vec administration and was surgically repaired and resolved. None of the reported SAEs led to persistent visual impairment. No ixo-vec related non-ocular TEAEs were reported. During the study, two participants died: one because of lung malignancy at 76 weeks and one from cardiopulmonary arrest due to hypoxia at 96 weeks; both deaths were unrelated to ixo-vec.

**Table 2:** Summary of treatment-emergent adverse events.

Treatment-Emergent Adverse Events	Ixo-vec 2 × 10^11^ (N = 15)	Ixo-vec 6 × 10^11^ (N = 15)
**Serious ocular TEAE, n (%)**	**1 (7%)**	**3 (20%)**
Cataract	1 (7%)	1 (7%)
Dry age-related macular degeneration^a^	1 (7%)	0 (0%)
Retinal detachment	0 (0%)	1 (7%)
Uveitis	0 (0%)	1 (7%)
**Serious non-ocular TEAE, n (%)**^b^	**4 (27%)**	**2 (13%)**
**APTC ATEs, n (%)**	**0 (0%)**	**0 (0%)**
Nonfatal MI	0 (0%)	0 (0%)
Nonfatal stroke	0 (0%)	0 (0%)
Vascular death	0 (0%)	0 (0%)
**Any TEAE of hypertension, n (%)**	**3 (20%)**	**0 (0%)**
**Ocular TEAEs in ≥10% of Study Participants (Study Eye), n (%)**		
Anterior Chamber Cell	7 (47%)	11 (73%)
Vitreal Cells	3 (20%)	8 (53%)
Anterior Chamber Flare	2 (13%)	7 (47%)
Conjunctival Haemorrhage	4 (27%)	5 (33%)
Keratic Precipitates	4 (27%)	5 (33%)
Vitreous Floaters	3 (20%)	6 (40%)
Iris Transillumination Defect	3 (20%)	5 (33%)
Posterior Capsule Opacification	5 (33%)	1 (7%)
Vitreous Haze	2 (13%)	4 (27%)
Anterior Chamber Pigmentation	3 (20%)	2 (13%)
Iris Adhesions	2 (13%)	2 (13%)
Iris Hyperpigmentation	2 (13%)	2 (13%)
Lenticular Pigmentation	1 (7%)	3 (20%)
Cataract	2 (13%)	1 (7%)
Dry Age-Related Macular Degeneration	3 (20%)	0 (0%)
Dry Eye	1 (7%)	2 (13%)
Iris Atrophy	1 (7%)	2 (13%)
Punctate Keratitis	2 (13%)	1 (7%)
Uveitis	0 (0%)	3 (20%)
Visual Acuity Reduced	2 (13%)	1 (7%)
Intraocular Pressure Increased	1 (7%)	2 (13%)

a Progression of dry age-related macular degeneration (dAMD) in one participant, not the de novo development of dAMD.

b Serious non-ocular TEAEs included stable angina pectoris (n = 1), cardiorespiratory arrest (n = 1), pneumonia (n = 2), sepsis (n = 1), fall (n = 1), intervertebral disc degeneration (n = 1), malignant lung neoplasm (n = 1), hemiparesis (n = 1), syncope (n = 1), acute kidney injury (n = 1), chronic obstructive pulmonary disease (n = 2), acute respiratory failure (n = 1), pneumothorax (n = 1), respiratory failure (n = 1), and hypertensive emergency (n = 1); AE, adverse event; APTC, Antiplatelet Trialists' Collaboration; ATE, arterial thromboembolic event; TEAE, treatment-emergent adverse event.

Ocular inflammation, as assessed by aqueous cell/flare and vitreous cell, was dose-dependent, mild to moderate and was responsive to topical corticosteroids. In some participants with inflammation asymptomatic pigment dispersion, iris transillumination defects and segmental iris atrophy were observed. There was no clinical or imaging evidence of chorioretinal inflammatory manifestations such as vasculitis, retinitis or choroiditis. There were also no cases of vascular occlusions or endophthalmitis. Frequency of inflammation (≥1+ AC or VC cells) was higher in the 6 × 10^11^ vg/eye dose group; it fluctuated but remained consistent over the course of the study (Figure S3). In the 2 × 10^11^ vg/eye dose group, frequency of inflammation decreased over time, and no participant had ≥1+ cells after week 24 (Figure S3). Past week 44, three participants had intermittent trace AC cells and two had intermittent trace VC cells. At study completion, zero participants (0%) in the 2 × 10^11^ vg/eye dose group and seven of 15 participants (47%) in the 6 × 10^11^ vg/eye dose group required topical corticosteroids for treatment of inflammation. Six participants of 15 (40%) in the 2 × 10^11^ vg/eye dose group and two participants of 15 (13%) in the 6 × 10^11^ vg/eye dose group did not require corticosteroid drops beyond the prophylaxis period.

### Secondary endpoints

**Fig. 2:**
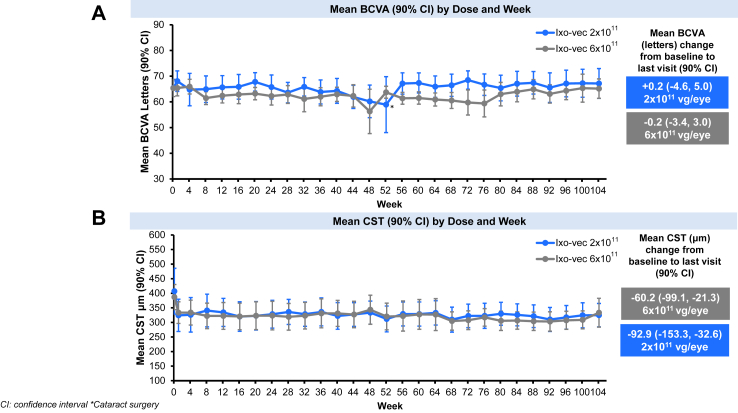
**A.** Mean Best Corrected Visual Acuity (BCVA ETDRS letters) (90% CI) by Dose and Week and Mean BCVA (ETDRS letters) change from baseline to last visit (90% CI). **B:** Mean Central Subfield Thickness (CST) μm (90% CI) by Dose and Week and Mean CST μm change from baseline to last visit (90% CI).

BCVA was maintained in both dose groups over 104 weeks. Mean change in BCVA from baseline was +0.2 letters in the 2 × 10^11^ vg/eye dose group and −0.2 letters in the 6 × 10^11^ vg/eye dose group (Fig. 2A). CST decreased following administration of aflibercept at screening and remained stable over 104 weeks in both dose groups. Mean CST change from baseline was −92.9 μm in the 2 × 10^11^ vg/eye dose group and −60.2 μm in the 6 × 10^11^ vg/eye dose group (Fig. 2B).

**Fig. 3:**
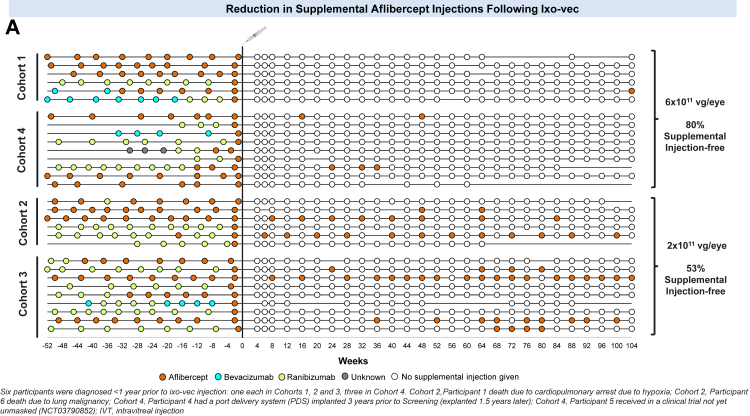

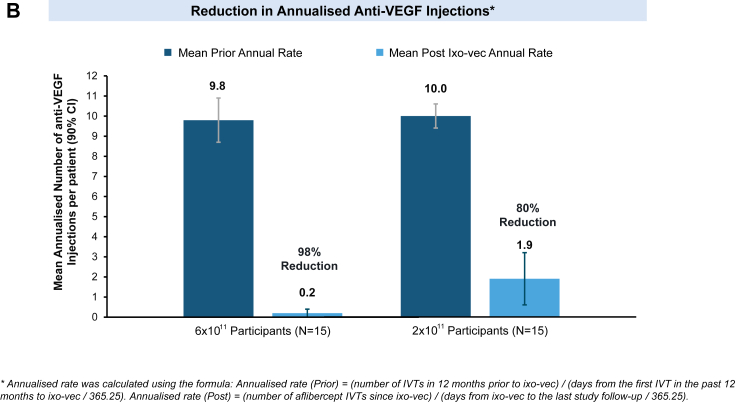
**A.** Reduction in supplemental aflibercept injections following ixo-vec. Each line represents individual participant data arranged by cohort and by dose. Historical anti-VEGF injections in the year prior to ixo-vec administration are shown on the left of the chart. On the right of the chart, open circles represent a study visit with no supplemental aflibercept injection administered; red circles represent a study visit where a supplemental aflibercept injection was administered. **B:** Reduction in Annualised Anti-VEGF Injection Frequency. Mean prior annualised number of anti-VEGF injection per patient (90% CI) compared to mean annualised number of anti-VEGF injection per patient post ixo-vec (90% CI). Annualised rate was calculated using the formula: Annualised rate (Prior) = (number of IVTs in 12 months prior to ixo-vec)/(days from the first IVT in the past 12 months to ixo-vec/365.25). Annualised rate (Post) = (number of aflibercept IVTs since ixo-vec)/(days from ixo-vec to the last study follow-up/365.25).

After ixo-vec administration, the frequency of anti-VEGF injections decreased in both dose groups. The mean annualised VEGF injections were reduced by 80% (10.0 mean annualised anti-VEGF injections to 1.9) in the 2 × 10^11^ vg/eye dose group and 98% (9.8 mean annualised anti-VEGF injections to 0.2) in the 6 × 10^11^ vg/eye dose group (Fig. 3B). At Week 104, 53% (8/15) of participants in the 2 × 10^11^ vg/eye dose group and 80% (12/15) in the 6 × 10^11^ vg/eye dose group were supplemental injection-free (Fig. 3A).

**Fig. 4:**
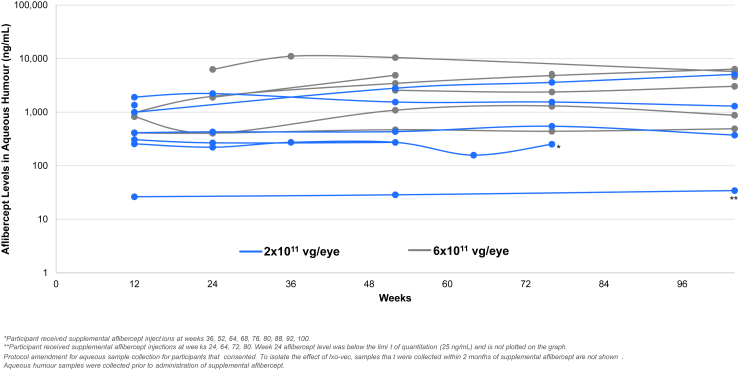
Aflibercept expression level in aqueous humour (ng/mL). Individual participant data is plotted for participants who consented. To isolate the effect of ixo-vec, samples that were collected within 2 months of a supplemental aflibercept injection are not shown. Aqueous humour samples were collected prior to administration of supplemental aflibercept.

Optional aqueous humour samples were collected in 17 participants. Measured aqueous aflibercept levels remained stable through the end of the study (Fig. 4). Participants with aflibercept levels >300 ng/mL did not require supplemental injections.

## Discussion

In this prospective study of 30 patients with nAMD, a single intravitreal injection of ixo-vec resulted in sustained therapeutic levels of intraocular aflibercept, improvement in retinal anatomy, maintenance of visual acuity, and a clinically meaningful reduction of injection frequency, with a majority of participants not requiring anti-VEGF rescue injections. Ixo-vec was generally well tolerated in this patient population. The most frequent adverse event was dose-dependent anterior chamber inflammation that was responsive to topical corticosteroids. To the best of our knowledge, ixo-vec is the first *in vivo* gene augmentation approach successfully administered intravitreally to deliver a standard-of-care therapeutic, aflibercept, targeting a prevalent, non-inherited ocular disorder, nAMD.

Previous unsuccessful efforts with *in vivo* gene therapy for nAMD have differed from ixo-vec in several ways. Intraocular delivery of ixo-vec results in the expression of aflibercept, a well-characterised, standard-of-care anti-VEGF therapy for nAMD.^4^ Other approaches have relied on transgenes (e.g., sFLT-1, sFLT01) which were never administered to patients to treat nAMD, and therefore the necessary therapeutic levels for efficacy were unknown.^18^^,^^19^ With ixo-vec, target intraocular levels could be calculated using the pharmacokinetic profile of the standard-of-care bolus injection.^4^^,^^8^^,^^12^ Additionally, the AAV2.7m8 capsid of ixo-vec was evolved in multiple species and selected for its robust transduction of retinal cells, including photoreceptors, ganglion cells, bipolar cells, optic nerve cells, Müller cells, ciliary epithelium and the iris pigment epithelium following intravitreal delivery.^4^^,^^16^^,^^17^^,^^20^ Prior programs have attempted intravitreal delivery with gene therapy vectors with known limited ability to cross the ILM and ocular transduction capacity (e.g., AAV2-sFLT01).^16^^,^^18^^,^^21^ In this study, aflibercept levels in aqueous humour were stable from the initial measurement at week 12 and maintained through study completion. Preclinical studies in nonhuman primates have demonstrated approximately 9-fold higher concentration of aflibercept in the retina and choroid compared to aqueous humour following IVT delivery of ixo-vec.^8^ As such, aqueous humour concentration of aflibercept is thought to be an indirect surrogate marker of a higher retina and choroid concentration, providing visual and anatomical stability while substantially reducing the need for anti-VEGF injections.

Ocular gene therapy can be delivered via subretinal, suprachoroidal or intravitreal routes.^2^^,^^21^^,^^22^ Most gene therapy programs for IRDs require the subretinal approach to adequately transduce the target cells of interest (i.e., retinal pigment epithelium transduction with voretigene neparvovec).^21^^,^^23^ Earlier nAMD gene therapy programs used subretinal delivery due to the limitations of prior vectors and the inability to cross the ILM when administered intravitreally (e.g., rAAV.sFLT-1; AAV8).^22^ However, with a widely prevalent disorder such as nAMD, and no requirement of cellular specificity for transduction nor protein production, the most desirable route of administration for gene therapy is intravitreal injection.^22^ Whereas subretinal delivery requires that patients undergo a pars plana vitrectomy in the operating room by a vitreoretinal surgeon, intravitreal injection of ixo-vec could be performed in office in the same manner as standard-of-care anti-VEGF treatments with similarly low procedural risks.^21^^,^^22^ Suprachoroidal delivery is a potential nonsurgical route of administration that can target affected chorioretinal tissues with limited impact on the anterior segment, but it requires further evaluation in clinical studies.^2^^,^^22^

The current nAMD therapeutic paradigm comes with a tremendous treatment burden of repeated intravitreal injections for the lifetime of the patient.^6^^,^^24^ Prior to receiving ixo-vec, patients in this study required a mean of nearly 10 annualised injections. Following *in-vivo* aflibercept gene augmentation, there was a noteworthy 80–98% reduction in the mean number of annualised injections and 53–80% of patients were free of injections. Ixo-vec maintained visual function with stable BCVA and improved retinal anatomy with significant decreases in OCT CST, while decreasing the intravitreal treatment burden. This is in direct contrast to current treatment paradigms where a decrease in the number of bolus anti-VEGF intravitreal injections results in potentially irreversible worsening of BCVA and OCT parameters.^14^^,^^25^^,^^26^

Although the eye is a relatively immune privileged organ, inflammation has nonetheless been a potentially limiting factor in ocular gene therapy.^26^, ^27^, ^28^, ^29^ Independent of the route of administration (subretinal, intravitreal, or suprachoroidal), a dose-dependent inflammatory reaction has been noted in nearly all ocular gene therapies.^18^^,^^28^ To mitigate this response, half the patients in this trial were prophylaxed with a short course of oral prednisone, the remainder with corticosteroid drops alone. Consistent with previous reports, patients receiving ixo-vec exhibited dose-dependent anterior chamber and vitreous inflammation. Some of these participants, both with phakic and pseudophakic eyes, exhibited asymptomatic pigment dispersion, iris transillumination defects, and segmental iris atrophy. These iris pigmentary changes may be associated with inflammation involving the iris/ciliary body. The mechanism of the iris pigmentary changes in the setting of intravitreal gene therapy and their clinical significance are currently unknown. Importantly, although some patients had evidence of vitreous inflammation, there was no evidence of chorioretinitis, including no retinal vasculitis or vascular occlusions. Inflammation was responsive to topical corticosteroid drops, and no patients in the 2 × 10^11^ vg/eye dose group required treatment by week 104. A superior safety profile together with comparable efficacy outcomes to the higher dose evaluated in this trial support continued study of ixo-vec 2 × 10^11^ vg/eye. The safety and efficacy of ixo-vec at 2 × 10^11^ vg/eye dose and a lower dose of 6 × 10^10^ vg/eye in combination with enhanced corticosteroid prophylaxis are being evaluated in the Phase 2 LUNA study in patients with nAMD (NCT05536973).

The OPTIC study has several limitations. As this was a Phase 1 trial, the sample size was limited to a total of 30 participants, with 15 per dose group. The sample size combined with some imbalances in baseline characteristics by cohort introduce the potential for bias, for example, patients who entered the study with high baseline CST would have had more potential for improvement in anatomical outcomes than those who had drier retinas at baseline. The study was unmasked and allowed for supplemental injections at investigator discretion following the initial protocol defined rescue; it is therefore possible that knowledge of dose level may have unintentionally impacted investigator decision to administer supplemental aflibercept treatment. The study had no comparator arm so direct comparison of ixo-vec to nAMD standard-of-care is not possible. It has been reported that some patients with nAMD do not require further treatment after discontinuation of anti-VEGF injections, therefore without a control arm we are unable to definitively conclude that participants in this study would have required additional therapy after ceasing standard-of-care injections.^30^^,^^31^ Due to the small sample size of each cohort, the effectiveness of the two corticosteroid regimens could not be compared. Another limitation of the study is that treat and extend and fixed regimens generally lead to increased frequency of anti-VEGF injections compared to PRN regimens based on retreatment criteria. Although we collected the number of injections in the 12 months prior to the study, we did not collect information on the specific regimens employed for each patient (PRN, treat and extend, or fixed dosing).

Creating cellular-based biofactories using ixo-vec offers a potential paradigm shift away from frequent anti-VEGF injections. This platform could be applied to other prevalent, chronic ocular diseases treated with repeated administration of a therapeutic protein. OPTIC participants will be followed for three additional years in an optional extension study to assess the long-term safety and efficacy of ixo-vec. Larger, randomised studies with longer follow-up are ongoing to fully address the therapeutic potential of ixo-vec and optimise the necessary anti-inflammatory prophylaxis.

## Contributors

AMK, CCW, DSB, SK, and AT participated in the design of the study. AMK, CCW, DSB, and SK participated in advisory committees. AMK, DSB, CCW, CDR, BGB, DP, CJD, BCJ, and JCM participated as study investigators. All authors participated in data acquisition or research execution, or both. All authors had access to study data and participated in data interpretation. AT and SK have verified the underlying data reported in the manuscript. SK participated in writing of the original draft. All authors participated in the writing and critical review of the manuscript and accept responsibility for the decision to submit for publication.

## Data sharing statement

Requests for access to anonymised patient data from this study can be submitted in writing to the corresponding author. Reasonable requests for data will be considered from researchers conducting approved clinical studies providing that the provision of such data does not violate any relevant patient confidentiality or data protection laws, or pose a commercial or legal conflict of interest. Data will be available after the study is completed, including any extension portion of the study. Proposals will be reviewed and approved by the sponsor on the basis of scientific merit. After approval of a proposal, a data access agreement must be signed after which data can be shared through a secure platform.

## Declaration of interests

**Arshad M Khanani, M.D., M.A.:**

• **Support of present manuscript**: Adverum Biotechnologies.
• **Consultant**: Adverum Biotechnologies, 4D Molecular Therapeutics, Genentech, Novartis, Regeneron Pharmaceuticals, Regenxbio,
• **Research Support**: Adverum Biotechnologies, 4D Molecular Therapeutics, Genentech, Novartis, Regenxbio.

**David S Boyer, M.D.**:

• 4D Molecular Therapeutics, Achillion, Adverum Biotechnologies, Alcon, Aldeyra Therapeutics, Alimera Sciences, Alkahest, Allegro, Allergan, Allgenesis, Amydis, Annexon Biosciences, Apellis Pharmaceuticals, Applied Genetec Technologies Crop (AGTC), Ashvattha, AsclepiX Therapeutics, Aviceda Therapeutics, Bausch & Lomb, Bayer, Biovisics Medical, Boehringer-Ingelheim, Cell Care Therapeutics, Chengdu Kanghong Biotechnology, Clearside Biomedical, Curacle Co LTD, Delsitech, Eyepoint Pharmaceuticals, Genentech, Glaukos, Iveric Bio, jCyte, Kriya Therapeutics, Kyowa Kirin, Lineage Cell, Lumithera, Inc, Nanoscope, NGM Biotherapeutics, Novartis, Ocular Therapeutix, Ocular Therapeutix, Ocugen, Oculis, Ocuphire, OcuTerra, Ocutrx Vision Technologies, Opthea, Optigo Biotechnology, Optos, Oxurion, Palatin, Pfizer, Regeneron Pharmaceuticals, RetinAI Medical AG, Ripple Therapeutics, Roche, Sanofi, Santen, Shenyang XingQi Pharma, Smilebiotek Zhuhai Limited, Stealth Biotherapeutics, Surrozen, Inc, Syneos, Thea Laboratories, UNITY Biotechnology, Vanotech, Verseon Corporation, Vitranu, Virto Biopharma, Viva Vision Biotech.

**Charles C Wykoff, M.D., Ph.D.**:

• **Consulting fees/honoraria paid to me for ongoing services provided as a Consultant for:** 4DMT, AbbVie, Adverum, Aerie, AGTC, Alcon, Alimera, Allergan, Allgenesis, Alnylam, Annexon, Apellis, Arrowhead, Bausch + Lomb, Bayer, Bionic Vision Technologies, Boehringer Ingelheim, Cholgene, Clearside, Curacle, Eyebiotech, EyePoint, Foresite, Frontera, Genentech, Gyroscope, IACTA, IVERIC Bio, Janssen, Kato, Kiora, Kodiak, Kriya, Merck, Nanoscope, Neurotech, NGM, Notal Vision, Novartis, OccuRx, Ocular Therapeutix, Ocuphire, Ocuterra, OliX, ONL, Opthea, Oxular, Palatin, PerceiveBio, Perfuse, PolyPhotonix, Ray, RecensMedical, Regeneron, RegenXBio, Resonance, Roche, Sandoz, Sanofi, SciNeuro, Stealth, Surrozen, Suzhou Raymon, Takeda, THEA, Therini, TissueGen, Valo, Verana.
• **Grants paid to my institution for ongoing Research support as a Principal Investigator for trials sponsored by:** 4DMT, Adverum, AffaMed, Alexion, Alimera, Alkahest, Allergan, Aldeyra, Allgenesis, Amgen, Annexin, Annexon, Apellis, Asclepix, Bayer, Boehringer Ingelheim, Chengdu Kanghong, Clearside, Curacle, Eyebiotech, EyePoint, Gemini, Genentech, GlaxoSmithKline, Graybug, Gyroscope, IONIS, iRENIX, IVERIC bio, Janssen, Kodiak, LMRI, McMaster University, Nanoscope, Neurotech, NGM, Novartis, Ocular Therapeutix, Ocuphire, OcuTerra, OliX, Ophthotech, Opthea, Oxurion, Oxular, Oyster Point, PerceiveBio, RecensMedical, Regeneron, RegenXBio, Rezolute, Roche, SamChunDang Pharm, Sandoz, Senju, Taiwain Liposome Co., UNITY, Verily, Xbrane.
• **Participation on a Data Safety Monitoring Board or Advisory Board: Kato, Aerie**.
• **Leadership or fiduciary role in other board, society, committee or advocacy group, paid or unpaid**: ASRS, Vit-Buckle Society.
• **Stock Options (not owner) from Private For-Profit Entities**: ONL, PolyPhotonix, RecensMedical, TissueGen, Visgenx, Vitranu.

**Carl D Regillo, M.D.**:

• **Consulting:** 4DMT, Adverum, Allergan, Annexon, Apellis, Aviceda, Bausch and Lomb, Clearside, Cognition, Eyepoint, Genentech, Iveric, Janssen, Kodiak, Lineage, Merck, NGM, Novartis, Ocugen, Ocular Therapeutics, Ocuterra, Ray, RegenXBio, Stealth, Thea, Zeiss.
• **Research Support:** Adverum, Allergan, Annexon, Apellis, Astellas, Eyepoint, Genentech, Gyroscope, Iveric, Janssen, Kodiak, Lineage, NGM, Notal, Novartis, Ocugen, Ocuterra, Opthea, Regeneron, RegenXBio.
• **Participation on a Data Safety Monitoring Board or Advisory Board**: Cognition, Iveric.

**Brandon G Busbee, M.D.**:

• **Consultant:** Genentech.
• **Speaker:** Apellis.
• **Royalties:** Thea.

**Dante Pieramici, M.D.**:

• **Medical writing support for present manuscript**: Adverum Biotechnologies.
• **Consultant**: Genentech, Regeneron, RegenXBio, Adverum, Eyepoint, Unity, Clearside, Gyroscope, Opthea.
• **Research Funding paid to institution**: Regeneron, RegenXBio, Adverum, Ocular Therapeutix, Eyepoint, Clearside, 4DMT, Janssen, Helios, Valo.
• **Participation on a Data Safety Monitoring Board or Advisory Board**: PERCEIVE
• **Leadership or fiduciary role in other board, society, committee or advocacy group, paid or unpaid**: Genentech.

**Carl J Danzig, M.D.**:

• **Research Grant:** Adverum, Genentech/Roche, Regeneron/Bayer, Kodiak, Unity, IvericBio, Gyroscope, Novartis, Curacle, Rezolute, 4DMT.
• **Consultant:** Adverum, Genentech/Roche, Regeneron/Bayer, Novartis, Kodiak, IvericBio.
• **Speaker:** Genentech, IvericBio, Novartis, Regeneron.
• **Advisory Board or Data Safety Monitoring Board:** Abbvie/RegenexBio, Genentech, Adverum.

**Brian C Joondeph, M.D.**:

• **Payment for expert testimony**: Contegrity Expert Group.
• **Leadership or fiduciary role in other board, society, committee or advocacy group, paid or unpaid**: Colorado Retina Executive Committee, unpaid.

**James C Major, M.D., Ph.D.**:

• **Research Support:** Adverum Biotechnologies, Inc.
• **Leadership or fiduciary role in other board, society, committee or advocacy group, paid or unpaid:** ASRS Board of Directors (unpaid).

**Adam Turpcu, Ph.D.**:

• Employee and stockholder of Adverum Biotechnologies, Inc.

**Szilárd Kiss, M.D.**:

• **Consultant/Advisor, Equity:** Adverum Biotechnologies
• **Consulting:** Regeneron, Optos, Novartis, Gyroscope, Apellis.
• **Advisory Board or Data Safety Monitoring Board:** Novartis.
• **Intellectual Property related to gene and cellular therapy:** Assigned to Weill Cornell/Cornell University.

## Appendix A Supplementary data

Supplementary Tables and Figures

advm-022-01 v8.0_OPTIC Protocol synopsis
