# Supplementary material for: Safety and efficacy of ixoberogene soroparvovec in neovascular age-related macular degeneration in the United States (OPTIC): a prospective, two-year, multicentre phase 1 study
Source: eClinicalMedicine. 2023 Dec 22;67:102394. doi: 10.1016/j.eclinm.2023.102394 (PMC10751837; doi:10.1016/j.eclinm.2023.102394)
Supplement: Supplementary Tables and Figures [file mmc1.pdf]

SUPPLEMENTARY APPENDIX

SUPPLEMENTAL FIGURES 2

Figure S1. Ixo-vec Platform 2

Figure S2. OPTIC Study Design. 3

Figure S3. Frequency of Inflammation Over 104 Weeks 4

SUPPLEMENTAL TABLE **5**

Table S1. TEAEs Related to Study Drug Per Investigator in >5% of Study Participants 5

SUPPLEMENTAL FIGURES

Figure S1. Ixo-vec Platform.

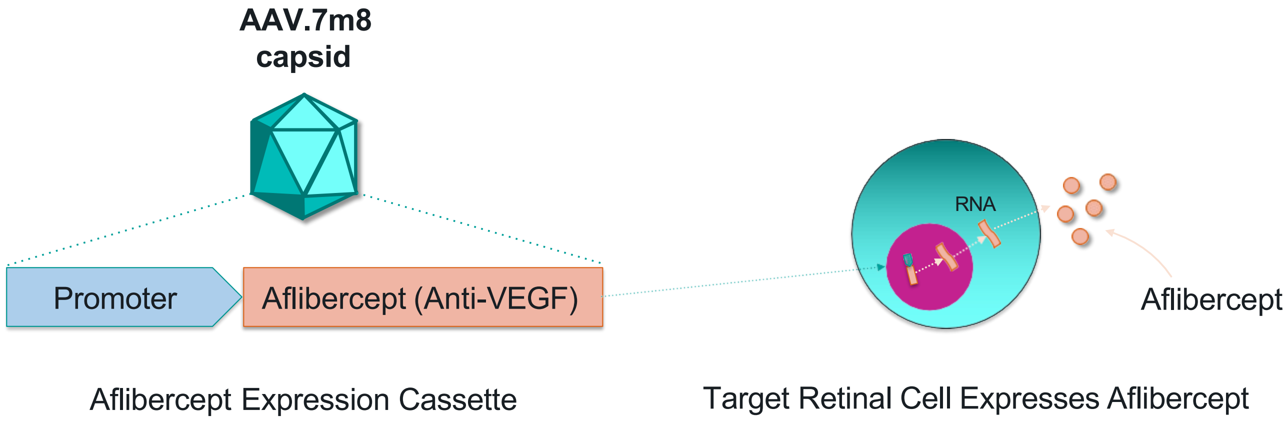

Figure adapted from Grishanin, R. et al. *Mol. Ther*. 2019;27:118–129; AAV, adeno-associated virus; cDNA, complementary deoxyribonucleic acid; RNA, ribonucleic acid; VEGF, vascular endothelial growth factor.

Figure S2. OPTIC Study Design.

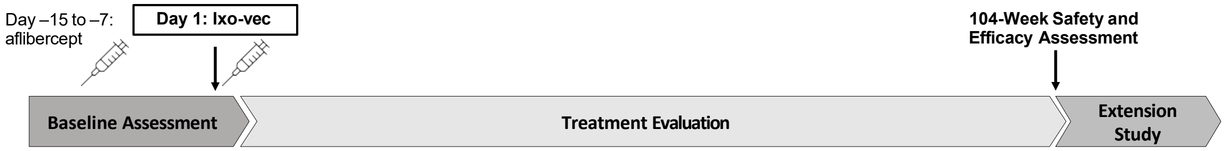

	**Prophylaxis Steroid Regimen**
**Cohort 1** (n=6) 6 x 10^{11} vg/eye high dose	Oral prednisone, 13d (60 mg for 6 days initiated 3 days prior to ixo-vec administration, followed by a 7-day taper)
**Cohort 2** (n=6) 2 x 10^{11} vg/eye low dose	Oral prednisone, 13d (60 mg for 6 days initiated 3 days prior to ixo-vec administration, followed by a 7-day taper)
**Cohort 3** (n=9) 2 x 10^{11} vg/eye low dose	Difluprednate, 6wks (QID for 3 weeks initiated on the day of ixo-vec administration, followed by a 3-week taper)
**Cohort 4** (n=9) 6 x 10^{11} vg/eye high dose	Difluprednate, 6wks (QID for 3 weeks initiated on the day of ixo-vec administration, followed by a 3-week taper)

QID, four times a day

Figure S3. Frequency of Inflammation Over 104 Weeks.

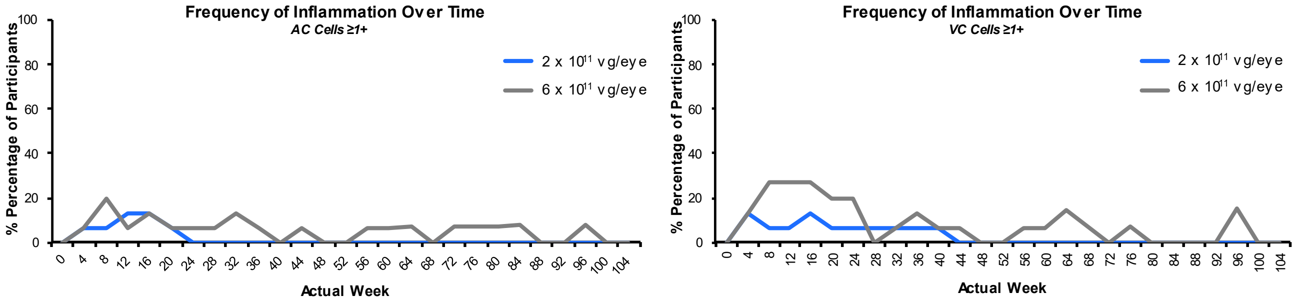

AC, aqueous cells; SAE, serious adverse event; VC, vitreous cells.

Cell grades as assessed by slit lamp, Grade categories are based on the Standardization of Uveitis Nomenclature (SUN) criteria for aqueous cells and National Institutes of Health guidelines for vitreous cells.

AC: 0.5+: 1-5 cells 1+: 6-15 cells 2+: 16-25 cells 3+: 26-50 cells 4+: >50 cells; VC: 0.5+: 1-10 cells 1+: 11-20 cells 2+: 21-30 cells 3+: 31-100 cells 4+: >100 cells; Rare cells are captured as 0.5+ for this analysis.

SUPPLEMENTAL TABLE

Table S1. TEAEs Related to Study Drug Per Investigator in >5% of Study Participants.

	**Ixo-vec $2x10^{11}$ (N=15)**	**Ixo-vec $6x10^{11}$ (N=15)**
**Ocular TEAEs Related to Study Drug Per Investigator in >5% of Study Participants, n (%)**		
Anterior Chamber Cell	6 (40%)	10 (67%)
Vitreal Cells	3 (20%)	8 (53%)
Anterior Chamber Flare	2 (13%)	7 (47%)
Keratic Precipitates	4 (27%)	5 (33%)
Iris Transillumination Defect	2 (13%)	5 (33%)
Vitreous Haze	2 (13%)	4 (27%)
Vitreous Floaters	0 (0%)	5 (33%)
Anterior Chamber Pigmentation	2 (13%)	2 (13%)
Iris Adhesions	2 (13%)	2 (13%)
Iris Hyperpigmentation	2 (13%)	2 (13%)
Iris Atrophy	1 (7%)	2 (13%)
Uveitis	0 (0%)	3 (20%)
Anterior Chamber Inflammation	1 (7%)	1 (7%)
Lenticular Pigmentation	1 (7%)	1 (7%)
Pupil Fixed	0 (0%)	2 (13%)

TEAE, treatment-emergent adverse event
