# Supplementary material for: Safety and efficacy of ixoberogene soroparvovec in neovascular age-related macular degeneration in the United States (OPTIC): a prospective, two-year, multicentre phase 1 study
Source: eClinicalMedicine. 2023 Dec 22;67:102394. doi: 10.1016/j.eclinm.2023.102394 (PMC10751837; doi:10.1016/j.eclinm.2023.102394)
Supplement: advm-022-01 v8.0_OPTIC Protocol synopsis [file mmc2.pdf]

CLINICAL STUDY PROTOCOL

Study ID	ADVM-022-01
Study Title	An Open Label Phase 1 Study of ADVM-022 (AAV.7m8-aflibercept) in Neovascular (Wet) Age-Related Macular Degeneration - [OPTIC]
Indication Studied	Neovascular (Wet) Age-Related Macular Degeneration
Sponsor	Adverum Biotechnologies, Inc.<br>800 Saginaw Drive, Redwood City, CA 94063
Protocol Version	Document Identifier/ Effective Date
Original	ADVM-022-01 / June 27th 2018
Amendment 1.0	ADVM-022-01 v1.0 / August 21st 2018
Amendment 1.1	ADVM-022-01 v1.1 / September 13th 2018
Amendment 2.0	ADVM-022-01 v2.0 / November 12th 2018
Amendment 3.0	ADVM-022-01 v3.0 / January 31st 2019
Amendment 3.1	ADVM-022-01 v3.1 / May 6th 2019
Amendment 4.0	ADVM-022-01 v4.0 / May 14th 2019
Amendment 4.1	ADVM-022-01 v4.1 / July 1st 2019
Amendment 5.0	ADVM-022-01 v5.0 / September 6th 2019
Amendment 6.0	ADVM-022-01 v6.0 / January 29th 2021
Amendment 7.0	ADMV-022-01 v7.0 / May 11th 2021
Amendment 8.0	ADVM-022-01 v8.0/ Sep 9th 2021

Confidentiality Statement

This document contains confidential and proprietary information of Adverum Biotechnologies, Inc. that must not be disclosed to anyone other than the recipient, study staff, and members of the Institutional Review Board/Ethics Committee. This information cannot be used for any other purpose other than the evaluation or conduct of the clinical study without the prior written consent of Adverum Biotechnologies Inc.

PROTOCOL APPROVAL—ADVERUM SIGNATORY

Study ID	ADVM-022-01
Study Title	An Open Label Phase 1 Study of ADVM-022 (AAV.7m8-aflibercept) in Neovascular (Wet) Age-Related Macular Degeneration - [OPTIC]
Version	Amendment 8.0, Sep 9th 2021

Study ADVM-022-01 will be conducted in accordance with the International Council for Harmonisation of Technical Requirements for Pharmaceuticals for Human Use (ICH) and Harmonised Tripartite Guidelines for Good Clinical Practices (E6), with applicable local regulations (including US Code of Federal Regulations [CFR] Title 21, and the ethical principles outlined in the Declaration of Helsinki).

Protocol approved by:

[redacted]
Chief Medical Officer
Adverum Biotechnologies, Inc.

Signature: [redacted]

Date: 12 Sep 2021

INVESTIGATOR AGREEMENT

Study ID	ADVM-022-01
Study Title	An Open Label Phase 1 Study of ADVM-022 (AAV.7m8-aflibercept) in Neovascular (Wet) Age-Related Macular Degeneration – [OPTIC]
Version	Amendment 8.0, Sep 9th 2021

I have read this clinical protocol as set forth below. On behalf of myself and the study staff, I agree to conduct Study ADVM-022-01 in compliance with the terms of the protocol as outlined herein and in compliance with Good Clinical Practice (GCP) and all applicable legal and regulatory requirements. Furthermore, I understand that the Sponsor, Adverum Biotechnologies, Inc, and the Institutional Review Board/Institutional Biosafety Committee (IRB/IBC) must approve any changes to the protocol in writing before implementation, except where it may be necessary to eliminate an immediate hazard to a subject enrolled in this study. I agree not to divulge to anyone, either during or after the study, any confidential information acquired regarding the investigational medicinal product and processes or methods of Adverum. All data pertaining to this study will be provided to Adverum.

____ ____________________________

Principal Investigator (signature) Date

Printed Name:

Institution:

STUDY CONTACTS

**Sponsor**	Adverum Biotechnologies, Inc.<br>800 Saginaw Drive, Redwood City, CA 94063
**Sponsor's Medical Monitor**	[redacted]<br>Chief Medical Officer<br>phone: [redacted]<br>email: [redacted]
**SAE Reporting**	phone: [redacted]<br>fax: [redacted]<br>emai[redacted]

1.0 STUDY SYNOPSIS

**Title of Study:**	An Open Label Phase 1 Study of ADVM-022 (AAV.7m8-aflibercept) in Neovascular (Wet) Age-Related Macular Degeneration –[OPTIC]
**Sponsor:**	Adverum Biotechnologies, Inc.
**Phase of Development:**	Phase 1
**Study Sites:**	Up to 10 sites in the United States
**Objectives:**	**Primary Objective**<br>• To assess the safety and tolerability of a single intravitreal (IVT) injection of ADVM-022<br>**Secondary Objectives**<br>• To evaluate the effect of ADVM-022 on Best Corrected Visual Acuity (BCVA)<br>• To evaluate the effect of ADVM-022 on central subfield thickness (CST) and macular volume<br>• To assess the need for rescue therapy<br>• To evaluate the effect of ADVM-022 on the presence of intraretinal fluid (IRF) and subretinal fluid (SRF)
**Primary Endpoint:**	Type, severity, and incidence of ocular and systemic adverse events (AEs)
**Secondary Endpoints**:<br>(Baseline measurements are based on values at Screening with Eylea injection):	• Mean change in BCVA from Baseline over time<br>• Percentage of subjects with a BCVA gain of ≥15 ETDRS letters from Baseline over time<br>• Percentage of subjects with a BCVA decline of ≤15 ETDRS letters from Baseline over time<br>• Mean change in CST and macular volume from Baseline over time<br>• Mean number of aflibercept injections over time<br>• Percentage of subjects requiring aflibercept injections over time<br>• Percentage of subjects without IRF over time<br>• Percentage of subjects without SRF over time

**Safety Assessments:**	The safety of ADVM-022 will be assessed through the collection of AEs, vital signs, physical and eye examinations, ECG, pregnancy testing, and laboratory evaluations.
**Efficacy Assessments:**	• BCVA as measured using ETDRS visual acuity chart<br>• Central subfield thickness as measured by Spectral Domain Optical Coherence Tomography (SD-OCT)<br>• Number of aflibercept injections post ADVM-022 administration<br>• Presence of IRF<br>• Presence of SRF
**Investigational Medicinal Product:**	ADVM-022 (AAV.7m8-aflibercept) is a genetically engineered, replication incompetent, capsid variant of the adeno-associated viral vector of serotype 2 (AAV2).<br><br>[redacted]
**Study Design:**	The Phase 1 study is designed to evaluate the safety, tolerability, and efficacy of a single IVT injection of ADVM-022. This study will enroll up to 30 subjects who are diagnosed with Neovascular (Wet) Age-Related Macular Degeneration (nAMD) and require regular IVT anti-VEGF therapy. Eligible subjects will be enrolled into one of four cohorts (6 subjects each in Cohorts 1 and 2 and 9 subjects each in Cohorts 3 and 4).<br><br>Cohort 1: ADVM-022 6×10^{11} vg/eye with prophylactic oral prednisone regimen<br><br>Cohort 2: ADVM-022 2×10^{11} vg/eye with prophylactic oral prednisone regimen<br><br>Cohort 3: ADVM-022 2×10^{11} vg/eye with prophylactic topical difluprednate regimen<br><br>Cohort 4: ADVM-022 6×10^{11} vg/eye with prophylactic topical difluprednate regimen<br><br>During Screening (Days -15 to -7), subjects with active nAMD will receive a single dose of aflibercept 2 mg IVT consistent with

	standard of care. As part of Screening, subjects will undergo clinical evaluation that includes SD-OCT 7 to 15 days after aflibercept administration to assess anti-VEGF response.<br><br>VEGF responsiveness will be confirmed by the Investigator and by the Sponsor for purposes of confirmation of anti-VEGF response at Day 1 visit prior to dosing with ADVM-022. Only those subjects confirmed by the Investigator and the Sponsor to have a meaningful anti-VEGF response will be eligible for this study. Subjects determined not to have a meaningful anti-VEGF response will have failed screening and will not be enrolled in this study.<br><br>A meaningful anti-VEGF response is defined as:<br><br>• Reduction from initial diagnosis in central subfield thickness by ≥ 30% as assessed using SD-OCT, **or**<br><br>• Reduction from screening in central subfield thickness by ≥ 20% as assessed using SD-OCT, **or**<br><br>• Normalization of CST with no wet AMD activity<br><br>Eligible subjects will receive ADVM-022 IVT administration on Day 1. Subjects will return on Days 3 and 8, and Weeks 2, 4, 6, and 8, and every 4 weeks thereafter, for clinical evaluation.<br><br>In Cohorts 1 and 2, subjects will be administered a prophylactic 13-day oral corticosteroid regimen, starting with 60 mg of prednisone 3 days before and 3 days after ADVM-022 treatment for a total of 6 days. This will be followed by a 7-day prednisone taper (Section 5.7.1). In Cohorts 3 and 4, subjects will be administered a prophylactic 6-week topical corticosteroid regimen of difluprednate starting after ADVM-022 administration (Section 5.7.2)<br><br>[redacted]

	[redacted]

	[redacted]<br><br>Starting on Week 4, subjects may receive rescue aflibercept 2 mg IVT if they meet the retreatment criteria. The presence of any one of the following shall warrant resumption of standard anti-VEGF treatment with aflibercept:<br><br>• Loss of ≥ 10 letters in BCVA (using the ETDRS protocol) from Baseline with intraretinal or subretinal fluid observed by SD-OCT and judged by the Investigator to be the cause of the BCVA loss<br>• An increase in central subfield thickness > 75 µm from Baseline as assessed by SD-OCT<br>• Presence of vision-threatening hemorrhage due to macular degeneration<br><br>**Independent Data Monitoring Committee (DMC):**<br><br>A review of safety data by the independent DMC will be performed approximately 4 weeks after the last subject in each cohort has received ADVM-022. The DMC will review and evaluate cumulative safety data to ensure the safety of trial subjects and provide recommendations to Sponsor to modify, temporarily suspend or terminate the trial if, following review of the data, there are safety concerns.
**Study Duration:**	Subject Participation: Duration of subject participation in the study will be approximately 108 weeks for each subject. This includes a screening period of up to 4 weeks and an additional 104-week study period.
**Number of Subjects:**	Up to 30 subjects
**Study Population:**	Subjects who are diagnosed with active CNV secondary to age-related macular degeneration (AMD) and a history of recent responsiveness to anti-VEGF treatment.

**Selection Criteria:**	Subjects must meet the following criteria to be included in this study. The subject must have diagnosis of recurrent and persistent nAMD that is responsive to anti-VEGF therapy and require frequent anti-VEGF injections to be potentially eligible for this study.<br>In addition to the Investigator, the Central Reading Center will review imaging as part of eligibility assessment. Confirmation of enrollment will be made by the Sponsor.
**Inclusion Criteria (at time of Screening):**	1) Male or female subjects, age ≥ 50 years of age<br>2) The study eye must have had prior or current evidence of active subfoveal CNV secondary to AMD occupying ≥ 50% of total lesion size with:<br>a) Leakage on fluorescein angiogram (FA), fluid on SD-OCT, or subretinal hemorrhage on color fundus photo<br>b) The entire dimension of the lesion must not exceed 12 Macular Photocoagulation Study disc areas<br>3) Subjects must be under active anti-VEGF treatment for nAMD with a minimum of 2 injections within 4 months prior to Screening.<br>4) Vision of study eye at Screening visit (prior to aflibercept injection):<br>a) BCVA 78 to 25 ETDRS letters, inclusive (approximate Snellen equivalent 20/32 to 20/320)<br>5) Vision of non-study eye:<br>a) BCVA of 35 ETDRS letters or more (approximate Snellen equivalent of 20/200 or better)<br>6) Demonstrate a meaningful anti-VEGF response as confirmed by the Investigator and the Sponsor and defined as:<br>a) Reduction from initial diagnosis in central subfield thickness by ≥ 30% as assessed using SD-OCT, **or**<br>b) Reduction from screening in central subfield thickness by ≥ 20% as assessed using SD-OCT, **or**<br>c) Normalization of CST with no nAMD activity<br>7) For men and women of childbearing potential, agreement to use an appropriate form of contraception for at least 12 weeks after the investigational medicinal product administration:

	a) Men will be required to use a contraceptive method (use of male condom with spermicide) to ensure that pregnancy is avoided in their female partners, unless a successful vasectomy (surgical male sterilization) has been performed<br>b) Women of childbearing potential must agree to use hormonal contraception, intrauterine device [IUD], tubal ligation, use of a male or female condom with spermicide, cervical cap or contraceptive sponge with spermicide, implantable or injectable contraceptives, or practice complete abstinence<br>8) Subjects must be able to provide written consent to participate in the study requirements and visits prior to any study procedures
**Exclusion Criteria:**	**Neutralizing Antibodies**<br>1) Documented anti-AAV.7m8 neutralizing antibody titer levels > 1:125 within 6 months prior to dosing with ADVM-022<br><br>**CNV Lesion**<br>2) Known history or evidence of the following CNV lesion characteristics:<br>a) Fibrosis or atrophy, retinal epithelial tear in the center of the fovea in the study eye, or any condition preventing visual acuity improvement<br>b) Scarring or fibrosis making up > 50% of total lesion area<br>c) Lesion size > 12 Macular Photocoagulation Study disc areas (30.5 mm^2), including blood, scars, and neovascularization as assessed by FA<br>d) Subretinal hemorrhage that is ≥ 50% of the total lesion area, or the presence of blood under the fovea that is ≥ 1 disc area in size in the study eye (if blood is under the fovea, then the fovea must be surrounded 270 degrees by visible CNV)<br><br>**Ocular Conditions (Retina/Posterior Segment)**<br>3) Significant epiretinal membrane or vitreomacular traction (VMT) syndrome in the study eye at time of dosing with ADVM-022 or history of a full thickness macular hole (Gass Stage 2 and above) in the study eye

	4) History of retinal disease in the study eye other than nAMD, including diabetic retinopathy (in either eye), retinal vein occlusion, uveitis, suspected retinal angiomatous proliferation, polypoidal choroidopathy, or CNV due to other causes (e.g., ocular histoplasmosis, trauma, or pathologic myopia), or any other vascular disease in the eye (benign conditions of the vitreous or peripheral retina are non-exclusionary)<br>5) History of retinal detachment (with or without repair) in the study eye<br>**Ocular Conditions (Non-retinal)**<br>6) Known history or evidence of significant non-retinal disease or media opacity in the study eye that could compromise vision during the course of the study, require surgery and/or preclude proper visualization or imaging of the retina (e.g., central corneal scarring, significant cataract, corneal dystrophy, scleromalacia)<br>7) Uncontrolled ocular hypertension or glaucoma in the study eye at time of dosing with ADVM-022 (defined as intraocular pressure [IOP] > 22 mmHg despite treatment with anti-glaucoma medication) or current use of > 2 IOP lowering medications<br>8) Known history or evidence of IOP elevation subsequent to steroid treatment (defined as an IOP > 22mmHg or use of any IOP lowering medication within 30 days of receiving a topical, periocular, IVT, or systemic steroid)<br>9) Active or history of ocular or periocular infection in either eye within 4 weeks prior to dosing with ADVM-022<br>**Ocular Surgeries/Procedures**<br>10) Any previous intraocular or periocular surgery on the study eye within 6 months of dosing with ADVM-022, or any planned major surgical procedure within 6 months of dosing with ADVM-022. Lid surgery > 1 month of dosing with ADVM-022 is not an exclusion<br>11) History of vitrectomy, trabeculectomy, or other filtration surgery in the study eye<br>12) YAG posterior capsulotomy within 3 months prior to dosing with ADVM-022<br>13) Any prior treatment with photodynamic therapy or retinal laser for the treatment of nAMD and any previous therapeutic radiation in the region of the study eye<br>**General/Systemic Conditions**

	14) History or evidence of any of the following cardiovascular disease within 6 months of dosing unless specified:<br>a) Severe cardiac disease (e.g., New York Heart Association [NYHA] Functional Class III or IV) history or clinical evidence of unstable angina<br>b) Acute coronary syndrome, myocardial infarction or coronary artery revascularization<br>c) Ventricular tachyarrhythmias requiring ongoing treatment, or uncontrolled arrhythmia<br>d) Uncontrolled hypertension defined as average systolic blood pressure (SBP) ≥160 mmHg or an average diastolic blood pressure (DBP) ≥100 mmHg, despite using BP–lowering medication within the screening period prior to dosing<br>e) History of cerebrovascular accident or transient ischemic attack<br>15) Any history of ongoing bleeding disorders or international normalized ratio (INR) >3.0. The use of aspirin or other anticoagulants (e.g., Factor Xa inhibitors) is not an exclusion. Note: INR can be repeated during the Screening period to confirm eligibility criteria are met.<br>16) Evidence of uncontrolled diabetes with a HbA1c >7.0% within the screening period prior to dosing with ADVM-022<br>17) History of malignancy within the last 5 years except for the following adequately treated<br>a) Local basal cell or squamous cell carcinoma of the skin<br>b) Carcinoma *in situ* of the cervix or breast<br>c) Papillary, noninvasive bladder cancer<br>d) Prostate cancer Stage 1 and 2 for which observation is clinically indicated with stable prostate-specific antigen (PSA) for 6 months<br>e) Any other cancer that has been in complete remission for at least 2 years or considered surgically cured<br>18) Positive HIV, Hepatitis B, or Hepatitis C (unless treated with a documented cure)<br>19) Within 36 hours prior to dosing with ADVM-022, evidence or suspicion of systemic active infection of any type deemed clinically significant by the Investigator based on clinical exam and/or temperature >38.5°C<br>20) Known serious allergies to:<br>a) Fluorescein dye or sodium fluorescein used in angiography (mild allergy amenable to treatment is allowable) or

	b) Aflibercept<br>21) Women who are pregnant, breastfeeding, or intend to become pregnant during the study<br>22) Other significant laboratory abnormalities or medical condition that the Investigator feels may compromise the subject's safety<br>**Medications**<br>23) Use of systemic anti-inflammatory steroids or immunosuppressant medications (other than protocol-specified prednisone) within 5 half-lives prior to dosing with ADVM-022. Inhaled or topical steroids and nonsteroidal anti-inflammatory drugs (NSAIDs) are permitted.<br>24) Received any:<br>a) Investigational medicinal product within 30 days or 5 half-lives prior to dosing with ADVM-022, whichever is longer<br>b) Prior gene therapy
**Method of Administration:**	Both ADVM-022 and aflibercept will be administered via IVT injection.<br>[redacted]
**Study Visits:**	Subjects will be enrolled into one of 4 cohorts.<br>Subjects will receive a single IVT injection of aflibercept 2 mg during Screening (Days -15 to -7).<br>Subjects will receive routine institutional standard post-injection care. On Day 1, subjects will receive ADVM-022 IVT injection. Subjects will return for clinical evaluation on Days 3 and 8, during Weeks 2, 4, 6, and 8, and every 4 weeks thereafter, until Week 104.<br>Starting on Week 4, subjects may receive rescue aflibercept 2 mg IVT if there is evidence of increased disease activity according to the retreatment criteria.
**Statistical Methods and Data Analysis:**	As a Phase 1 safety study, no formal statistical analysis is required. Descriptive and exploratory analyses are to be performed on all data to gain further insight into the safety and activity of this gene transfer therapy.
